# Combinatorial Inputs to the Ventral Striatum from the Temporal Cortex, Frontal Cortex, and Amygdala: Implications for Segmenting the Striatum

**DOI:** 10.1523/ENEURO.0392-17.2017

**Published:** 2017-12-22

**Authors:** Eun Young Choi, Song-Lin Ding, Suzanne N. Haber

**Affiliations:** 1 Department of Pharmacology and Physiology, University of Rochester Medical Center, Rochester, NY 14642; 2 Allen Institute for Brain Science, Seattle, WA 98109; 3 Institute of Neuroscience, School of Basic Medical Sciences, Guangzhou Medical University, Guangzhou, Guangdong Province 511436, P. R. China

**Keywords:** anatomic connections, basal ganglia, circuit integration, corticostriatal circuitry

## Abstract

The canonical striatal map, based predominantly on frontal corticostriatal projections, divides the striatum into ventromedial-limbic, central-association, and dorsolateral-motor territories. While this has been a useful heuristic, recent studies indicate that the striatum has a more complex topography when considering converging frontal and nonfrontal inputs from distributed cortical networks. The ventral striatum (VS) in particular is often ascribed a “limbic” role, but it receives diverse information, including motivation and emotion from deep brain structures, cognition from frontal cortex, and polysensory and mnemonic signals from temporal cortex. Using anatomical tract-tracing in 17 male adult monkeys (*Macaca nemestrina*, *Macaca fascicularis*, *Macaca mulatta*), we build upon this striatal map by systematically mapping inputs from frontal cortex, amygdala, temporal pole, and medial temporal cortex. We find that the VS contains heterogeneous subregions that become apparent when considering both the identities and strengths of inputs. We parcellated the VS into a ventromedial sector receiving motivation and emotion-related information from regions including area TG, ventromedial PFC, ACC, and amygdala; and a more functionally diverse dorsolateral sector that receives this information coupled to cognitive and sensorimotor information from dorsolateral PFC, ventrolateral PFC, premotor cortex, area TAr, and area TEr. Each sector was further parcellated into smaller regions that had different proportions of these inputs. Together, the striatum contains complex, selective input combinations, providing substrates for myriad associations. This VS parcellation provides a map that can guide and interpret functional interactions in healthy individuals and those with psychiatric disorders, and may be useful in targeting treatments for specific psychiatric conditions.

## Significance Statement

The striatum is central for learning associations among stimuli, actions, and outcomes. The canonical striatal map is divided into ventromedial-limbic, central-association, and dorsolateral-motor territories. However, the striatum has a more complex topography. We find that the ventral striatum (VS) consists of a ventromedial sector receiving motivation and emotion-related information and a more functionally diverse dorsolateral sector that receives this information coupled to cognitive and sensorimotor information from frontal cortex, temporal cortex, and amygdala. Each sector is further parcellated into smaller regions with different input proportions. These results show the complex, selective combinations of inputs to VS, providing an anatomic substrate for understanding how various functions may be integrated and a guide for interpreting striatal activity associated with different behavioral outcomes.

## Introduction

The canonical striatal functional map is composed of a ventromedial-to-dorsolateral gradient of frontal cortical projections (Haber, 2003). This gradient is broadly segmented into three distinct, but overlapping, functional territories: ventromedial-limbic, central-association, and dorsolateral-motor (Parent and Hazrati, 1995). Although this model has been a useful heuristic in understanding striatal function, recent findings demonstrate that the corticostriatal map is more complex. Indeed, the presence of connectional hubs, which are regions with a high convergence of diverse functional projections (Averbeck et al. 2014; Choi et al. 2017), suggests that parsing the striatum into three simple sections is not sufficient. Adding to the complexity, inputs from other cortical lobes (e.g., temporal, parietal lobes) overlap with frontal projections in the striatum (Yeterian and Van Hoesen, 1978; Selemon and Goldman-Rakic, 1985; Jarbo and Verstynen, 2015; Choi et al. 2017). Human resting-state functional connectivity MRI (fcMRI) demonstrates that such distributed cortical areas form large-scale cortical networks associated with different striatal regions (Di Martino et al. 2008; Barnes et al. 2010; Choi et al. 2012). Using the precision of anatomic tract-tracing, the present study systematically investigates the overlap of the functional map formed by frontal cortical and amygdala projections with temporal projections to the ventral striatum (VS), examining the interactions of these distributed cortical networks at the anatomic level in the striatum. Taken together, an updated striatal map identifying these combinations of inputs is critical for understanding the precise modification of information that occurs within a striatal region.

The VS, which includes the nucleus accumbens (NAc) core and shell and adjacent caudate and putamen, is often equated as the part of the striatum that is involved in motivation and reward. However, the VS is a large region, estimated to be 20%–25% of the striatum (Haber et al. 2006; Tziortzi et al. 2014). It receives inputs from reward-encoding neurons in the ventral tegmental area, inputs from the amygdala and ventromedial prefrontal cortex (vmPFC) that encode emotional valence, and motivation-related inputs from the orbitofrontal cortex (OFC) that are involved in binding stimulus to response. In addition, cognitive inputs from the dorsolateral and ventrolateral PFC (dlPFC, vlPFC), which are central for cognitive control, converge with these projections within the VS (Haber et al. 2006). Although less well understood, rostral and medial parts of the temporal cortex also project strongly to the VS (Van Hoesen et al. 1981; Webster et al. 1993; Friedman et al. 2002). These inputs provide polymodal auditory, visual, and visceromotor, as well as memory-related, information (Zola-Morgan et al. 1989; Tanaka et al. 1991; Meunier et al. 1993; Suzuki et al. 1993; Poremba et al. 2003; Olson et al. 2007), forming a network with the VS that is critical for integrating sensory and affective inputs with memories and behavioral goals to select and enact action plans.

The goal of this study was to build on the existing striatal map of PFC and amygdala inputs (Haber et al. 2012) by examining the combined inputs from the frontal cortex, amygdala, and temporal cortex to the VS. Here, we focus on heteromodal temporal polar cortex (TP) with adjacent visceromotor, auditory, and visual areas (TP+) and the medial temporal cortex (MTC), which plays a key role in memory (Zola-Morgan et al. 1989; Meunier et al. 1993; Suzuki et al. 1993). In contrast to striatal projections from the superior temporal areas, which primarily target the dorsal striatum (DS; Maunsell and Van Essen, 1983; Saint-Cyr et al. 1990; Yeterian and Pandya, 1997), less is known about how these rostral and medial temporal connections target the VS (Van Hoesen et al. 1981; Webster et al. 1993; Friedman et al. 2002). Here, using systematic anterograde and retrograde tracer injections, we explored, first, the full topography of TP+ and MTC projections to the VS and, second, their quantitative relationship to frontal cortical and amygdala inputs.

## Materials and Methods

### Experimental design and statistical analysis

**Figure 1.**
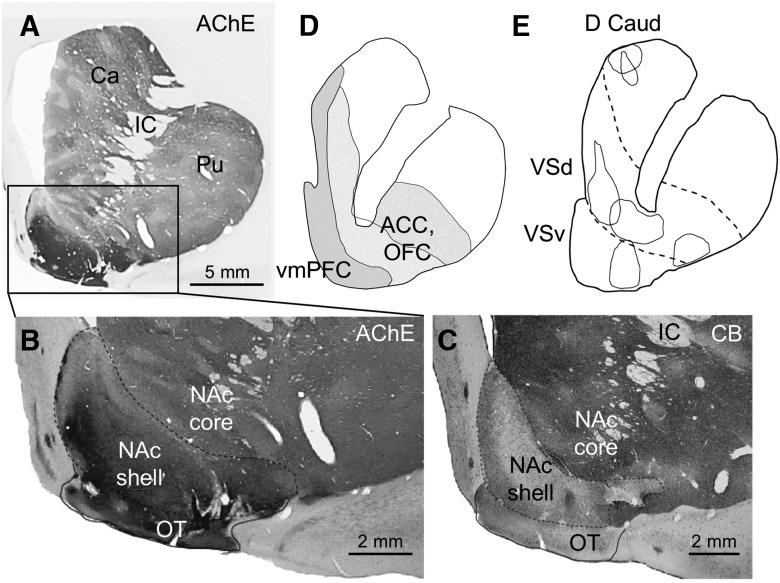
Ventral striatum. The boundary between the VSd and VSv and the ventral border of the VSv were based on AChE (***A***, ***B***) and CB (***C***) staining identifying the shell of the NAc and olfactory tubercle. ***D***, The dorsal border of the VSd was based on corticostriatal projection zones from emotion-processing prefrontal cortical regions (vmPFC, ACC, OFC). ***E***, Schematic of VSd and VSv boundaries (dashed lines) and six striatal retrograde injection sites. OT, olfactory tubercle; IC, internal capsule; Ca, caudate; Pu, putamen.

**Figure 2.**
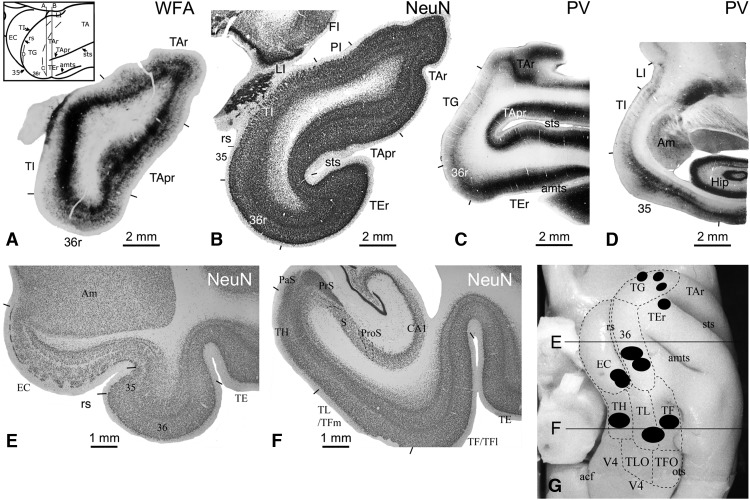
Temporal pole, adjacent areas, and medial temporal cortex. Coronal slices of areas and structures within TP+ stained with WFA (***A***) or NeuN (***B***). ***C***, ***D***, Sagittal slices of TP+ stained with PV. Slice locations for ***A–D*** shown in inset of ***A***. Coronal slices of the anterior MTC (***E***) and posterior MTC (***F***) stained with NeuN. ***G***, Orbital view of temporal lobe showing schematic of 11 anterograde injections placed in TP+ (area TG, TEr) and MTC (EC, 36, TH, TL/TF, TF). Lines indicate slice locations for ***E***, ***F***. Short black lines in ***A–F*** and dashed lines in ***G*** indicate areal boundaries. Am, amygdala; CA, cornu ammonis; Hip, hippocampus; LI, limen insula; PaS, parasubiculum; ProS, prosubiculum; S, subiculum; acf, anterior calcarine fissure; amts, anterior middle temporal sulcus; ots, occipital temporal sulcus; rs, rhinal sulcus; sts, superior temporal sulcus.

**Figure 3.**
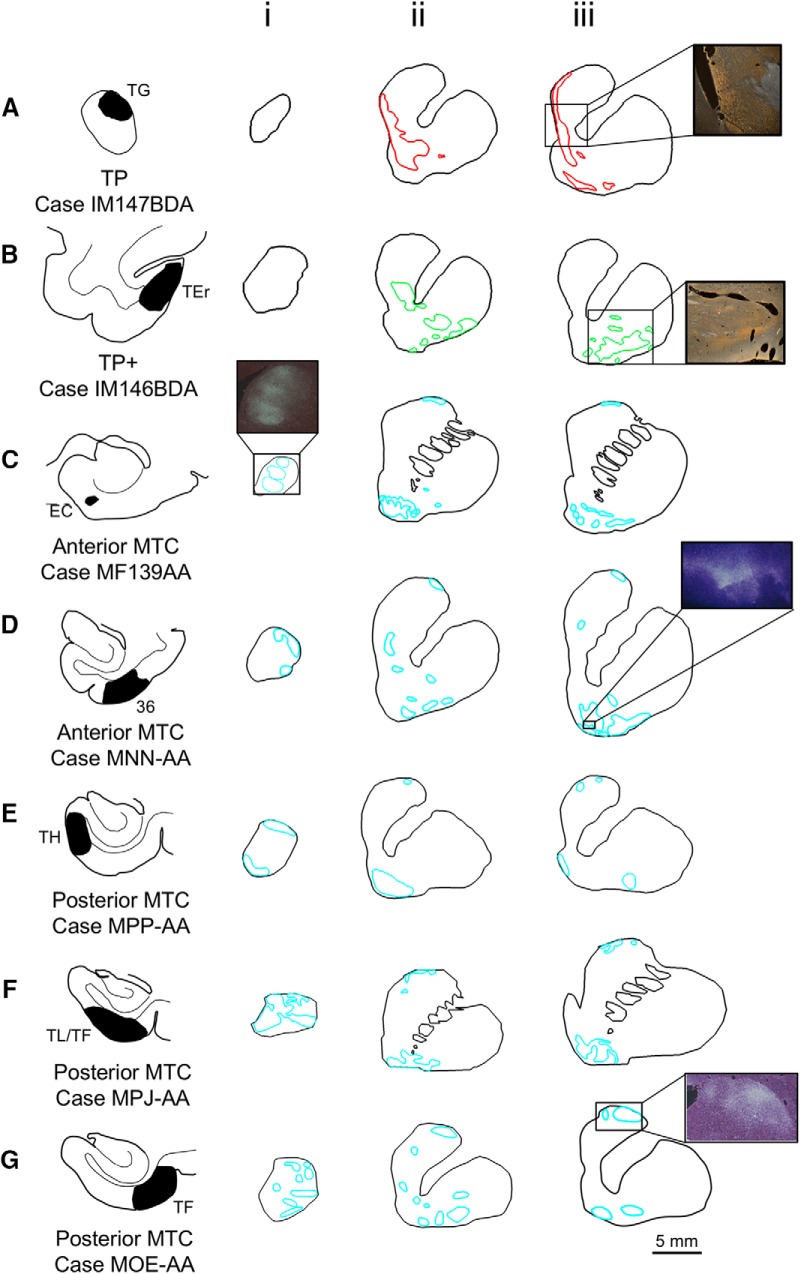
Temporal inputs to the striatum: anterograde labeling. Anterograde tracer injections in the TP (***A***), TEr in the rostral inferior temporal cortex (***B***), and the anterior (***C***, ***D***) and posterior (***E***, ***G***) MTC show three broad patterns (red, green, cyan) of dense terminal projections in the striatum. Note the split in projections from MTC to the dorsal and ventral striatum.

Eleven anterograde tracer injections were placed in TP+ and MTC: three in TG, two in area 36, one in TEr, two in EC, one in TH, one in TF, and one in TL (Table 1; Figs. 2*G* and 3). Dense terminal fields in the striatum were charted in one of eight coronal slices. To determine the overlap between terminals from the different temporal regions with those from the frontal cortex and amygdala, we first rendered the terminal fields for each case in 3-D. We then merged each into a reference model that also contained projections from the amygdala and previously reported projections from frontal cortex (Haber et al. 2006; Calzavara et al. 2007; Averbeck et al. 2014). Using retrograde tracers to specifically target regions of convergence, we confirmed and extended these findings by stereologically counting the number of TP+ and MTC cells projecting to each VS region. There were four retrograde tracer injections located in the medial, ventral, central, and lateral regions of the VSd and VSv (Table 1; Figs. 1*E* and 5). In addition, there were two retrograde tracer injection sites in the dorsal caudate nucleus area that received projections from the MTC (Table 1; Figs. 1*E* and 5). Finally, we determined the relative strengths of the projections from each frontal and temporal area in each VS case and one of the dorsal caudate cases by calculating the percentage of labeled cells located within that area from the total number of labeled cells identified in frontal and temporal cortex. Differences in the input patterns were assessed with pairwise linear correlations (*r*) of the percents of inputs from all examined areas for each pair of injections. Two-tailed *p*-values were obtained with Fisher’s *r*-to-*t* transformation and Student’s *t*-distribution.

**Table 1.** Injection cases

Case	Species	Tracer (direction analyzed)	Injection site
IM143	*Macaca fascicularis*	BDA (anterograde)	Temporal pole: area TG
IM145	*Macaca fascicularis*	BDA (anterograde)	Temporal pole: area TG
IM147	*Macaca fascicularis*	BDA (anterograde)	Temporal pole: area TG
IM146	*Macaca fascicularis*	BDA (anterograde)	Temporal pole+: area TEr
MF139	*Macaca fascicularis*	[^3^H]AA (anterograde)	Anterior MTC: entorhinal cortex
MME	*Macaca mulatta*	[^3^H]AA (anterograde)	Anterior MTC: entorhinal cortex
MNN	*Macaca mulatta*	[^3^H]AA (anterograde)	Anterior MTC: area 36
MPW	*Macaca mulatta*	[^3^H]AA (anterograde)	Anterior MTC: area 36
MPP	*Macaca mulatta*	[^3^H]AA (anterograde)	Posterior MTC: area TH
MPJ	*Macaca mulatta*	[^3^H]AA (anterograde)	Posterior MTC: area TL/TF
MOE	*Macaca mulatta*	[^3^H]AA (anterograde)	Posterior MTC: area TF
MF205	*Macaca fascicularis*	FS (anterograde)	Basolateral amygdala
MR28	*Macaca mulatta*	WGA (retrograde)	VSv (nucleus accumbens)
MN33	*Macaca nemestrina*	WGA (retrograde)	Medial VSd
MN38	*Macaca nemestrina*	LY (retrograde)	Central VSd
MN40	*Macaca nemestrina*	LY (retrograde)	Lateral VSd
MF170	*Macaca fascicularis*	FS (retrograde)	Medial dorsal caudate
MF184	*Macaca fascicularis*	WGA (retrograde)	Medial dorsal caudate

### Surgery and tissue preparation

Eighteen male adult macaque monkeys (three *Macaca nemestrina*, eight *Macaca fascicularis*, seven *Macaca mulatta*) received tracer injections. All experiments were conducted in accordance with the *Guide for the Care and Use of Laboratory Animals* (Institute of Laboratory Animal Resources, National Research Council, 1996) and were approved by the University of Rochester’s University Committee on Animal Resources or by the University of Iowa’s Committee on Animal Care and Use. The details of the surgical procedures and histologic processing have been previously described (Ding et al. 2000, 2009; Haber et al. 2006). Briefly, monkeys received an injection of one or more of the following anterograde, retrograde, or bidirectional tracers: biotinylated dextran amine (BDA; Invitrogen), Lucifer yellow (LY) or fluorescein (FS) conjugated to dextran amine (Invitrogen), *Phaseolus vulgaris* leucoagglutinin (PHA-L; Vector Laboratories), wheat germ agglutinin-horseradish peroxidase (WGA; Sigma-Aldrich), or tritiated amino acids ([^3^H]AA; 1:1 solution of [^3^H]leucine and [^3^H]proline, NEN; see Table 1). Twelve to fourteen days after the operation, monkeys were deeply anesthetized, and brains were perfused with saline and paraformaldehyde and sectioned at 50 μm. Immunocytochemistry was performed on one in every eight (0.4-mm interval) free-floating sections. Tissue was incubated with primary anti-LY (1:3000 dilution; Invitrogen), anti-FS (1:1000; Invitrogen), anti-PHAL (1:2000; EY Laboratories), or anti-WGA (1:50,000, Sigma-Aldrich), a biotinylated secondary antibody, followed by an avidin-biotin complex (ABC) solution (Vectastain ABC kit; Vector Laboratories). BDA injection cases were treated only with the ABC solution. For all cases, immunoreactivity was visualized using standard DAB procedures. For autoradiography, one of every eight sections (0.4-mm interval) were exposed for 4-6 months at 4°C in a Kodak NTB2 photographic emulsion in a light-tight box. Sections were developed in Kodak D19, fixed, and counterstained with cresyl violet. Cortical cytoarchitectonic boundaries were determined with combinations of a cresyl violet stain for Nissl substance, *Wisteria floribunda* agglutinin (WFA; Sigma-Aldrich), AChE (1:10,000; Sigma-Aldrich), PV (1:10,000; Swant), CB (1:10,000; Swant), and NeuN (1:1500; Millipore Bioscience Research Reagents).

### Microscopy

The distributions of labeled neurons and terminal fields were charted with light- and dark-field microscopy under 1.6×, 4×, 10×, 20×, and 40× objectives, as previously described (Haber et al. 2006; Choi et al. 2017). Briefly, for temporal and amygdala anterograde tracer injection cases, outlines of dense, focal projection patches comprised of fibers containing synaptic boutons were charted with Neurolucida software (MicroBrightField) throughout the rostrocaudal striatum in one of every eight tissue sections (0.4-mm interval). For striatal retrograde tracer injection cases, StereoInvestigator software (MicroBrightField) was used to obtain unbiased quantitative estimates of the labeled cells in cortical areas of the frontal cortex, TP+, and MTC in one of every 24 sections (1.2-mm interval). Cell counts were obtained in 31 areas of the frontal cortex, TP+, and MTC in four VS cases and one dorsal caudate case. Labeled cells in the amygdala were identified in one of every 24 sections (1.2-mm interval) with StereoInvestigator for cases MN33WGA and MF170FS. Cases MR28WGA, MN38LY, and MN40LY were previously published by Fudge et al. (2002) and had labeled cells that were marked by hand with camera lucida.

### Analysis

2-D charts of the terminal fields in the striatum from temporal anterograde tracer injection cases were rendered in 3-D and linearly transformed into a standard atlas of the striatum using IMOD (Boulder Laboratories). Temporal projection patterns were assessed both within coronal sections and globally across the striatum. Temporal projections were then overlaid with those from our library of previously reported anterograde tracer injections in the dlPFC (eight injection cases in areas 9 and 46), vlPFC (two injection cases in areas 44/45 and 47), ACC (four injection cases in area 24), OFC (eight injection cases in areas 11, 13, and 14), and premotor cortex (four injection cases in area 6; Haber et al. 2006; Calzavara et al. 2007; Averbeck et al. 2014), as well as a previously unpublished anterograde tracer injection in the basolateral nucleus of the amygdala. Areas of overlap between temporal, frontal, and amygdala projections were identified. To allow for the comparison of connectivity strengths across striatal retrograde injection cases, within each case, each cortical area’s percentage of the total cells in all the sampled frontal and temporal areas was determined. Differences in the input patterns were assessed with a pairwise linear correlation (*r*) of the input percentages for each pair of injections using the *corr* function in Matlab (Matlab R2017a, MathWorks). Two-tailed *p*-values testing against the null hypothesis of no correlation were obtained with the same Matlab function, which uses Fisher’s transformation to convert the *r* value into a *t* statistic with a Student’s *t*-distribution.

### Nomenclature and subdivisions

#### Ventral striatum

We divide the VS into dorsal and ventral parts. The ventral VS (VSv) consists of the shell of the NAc and the olfactory tubercle. The dorsal VS (VSd) consists of the core of the NAc and the adjacent caudate and putamen (Haber and McFarland, 1999; Haber et al. 2006). In general, the NAc shell and olfactory tubercle have densely packed neurons and contain relatively heavy acetylcholinesterase (AChE) staining (except the most mediodorsal portion; Fig. 1*A*, *B*) and light calbindin-D28k immunoreactivity (Fig. 1*C*; Meredith et al. 1996). In contrast, the VSd has less densely packed neurons, moderate AChE staining (Fig. 1*A*, *B*), and dense calbindin staining (Fig. 1*C*; Meredith et al. 1996). The dorsal border of the VSd is poorly defined by cyto- and chemo-architecture and thus was designated based on the zone that receives inputs from emotion-processing cortical regions—the vmPFC (areas 14, 25), OFC (areas 11, 13), and dorsal ACC (dACC; area 24; Haber et al. 2006; Fig 1*D*, *E*). This territory encompasses the medial caudate adjacent to the ventricle and the caudate–putamen ventral to and around the ventral end of the internal capsule.

#### Temporal pole+

Areas within TP+ were identified based on a parcellation of the human temporal polar cortex (Ding et al. 2009). TP refers specifically to area TG. TP+ consists of TG and the adjacent areas of TI, PI, and the rostral portions of the superior (areas TAr, TApr), inferior (TEr), and medial (EC, area 35r, area 36r) temporal cortices (Fig. 2*A*, *B*). TG is a dysgranular region at the tip of the temporal pole and is both parvalbumin (PV; Fig. 2*C*) and WFA positive, with relatively light staining. TI is a narrow agranular temporal insular region located rostrodorsally to the rhinal sulcus (Fig. 2*B*). TI has light PV staining (Fig. 2*D*) but dense WFA (Fig. 2*A*) staining. TApr is the rostral extension of the polysensory granular cortical region in the dorsal bank of the STS (Fig. 2*A–C*), an area included in TA by von Bonin and Bailey (1947). TApr has dense PV (Fig. 2*C*) and WFA (Fig. 2*A*) staining. TAr is the granular cortical region located rostral to the parabelt auditory association cortex (area TA) and caudal to area TG (Fig. 2*A–C*) and shows moderate PV (Fig. 2*C*) and WFA (Fig. 2*A*) labeling. TEr (Fig. 2*B*) is a granular region with heavy PV (Fig. 2*C*) and WFA labeling. In addition, PI, the anterior-most portion of the para-insular cortex (Fig. 2*B*), was identified as a dysgranular region with lighter PV and WFA staining among areas LI, TI, and TAr.

#### Medial temporal cortex

The parcellation of the MTC has been previously described in detail (Blatt et al. 2003; Suzuki and Amaral, 2003; Ding and Van Hoesen, 2010). The anterior MTC refers to the cortex adjacent to the amygdala and hippocampus at the coronal levels of the rhinal sulcus and includes the EC and perirhinal cortex (areas 35 and 36; Fig. 2*E*, *G*). The posterior MTC is composed of the parahippocampal cortex (areas TH, TL, TF, THO, TLO, and TFO; Fig. 2*F*, *G*).

#### Frontal cortex and amygdala

Boundaries of frontal cortical areas were identified as previously described (Haber et al. 2006; Choi et al. 2017) based on a combination of anatomic landmarks (gyri, sulci, white matter; Paxinos et al. 2000) and detailed cytoarchitectonic descriptions (Preuss and Goldman-Rakic, 1991; Vogt, 1993, 2009). Frontal areas were grouped into: frontal pole (area 10), vmPFC (areas 14, 25), OFC (areas 11, 13, OPAI, OPro), ACC (areas 32, 24), dlPFC (areas 9, 9/46, 46, 8), vlPFC (areas 44, 45, 47, ProM), and premotor cortex (area 6). The central, basal, and lateral nuclei of the amygdala were identified by Nissl, neuronal nuclear antigen (NeuN), and AChE staining as previously described (Price et al. 1987; Amaral and Bassett, 1989; Fudge et al. 2002).

## Results

Anterograde tracer injections in TG, TEr, and the entorhinal, perirhinal, and parahippocampal cortices of the MTC resulted in distinct corticostriatal projection patterns. TG projections terminated medially, while those from TEr terminated laterally in the VS. Interestingly, projections from the MTC bifurcated into those targeting the VS and those targeting the dorsal edge of the caudate. Overlay of these temporal projections with previously reported frontal inputs (Haber et al. 2006; Calzavara et al. 2007; Averbeck et al. 2014) and amygdala inputs indicated two distinct combinations of projections that parcellated the VS into ventromedial and dorsolateral sectors. Systematic retrograde tracer injections in the VS confirmed these projection patterns and identified the set of convergent temporal, frontal, and amygdala inputs to subregions in these VS sectors. Overall, the VSv and medial VSd mainly received inputs from cortical areas linked to reward, motivation, and visceromotor function, specifically the ventral and medial PFC (vmPFC, OFC, dACC), areas TG and TI in TP+, amygdala, and MTC. In contrast, while the central and lateral VSd also received these inputs, additional afferents also came from the lateral and dorsal PFC (dlPFC, vlPFC, dmPFC) and auditory and visual areas (TApr, TAr, TEr) of TP+. Within these broad patterns, each striatal region received a unique profile of inputs. These results show the specific combinations of temporal, PFC, and amygdala projections to the VS, creating unique striatal sectors that may underlie functional integration and parcellation in the VS.

### Temporal polar and medial temporal projections to the ventral striatum

#### Anterograde tracer injections

**Figure 4.**
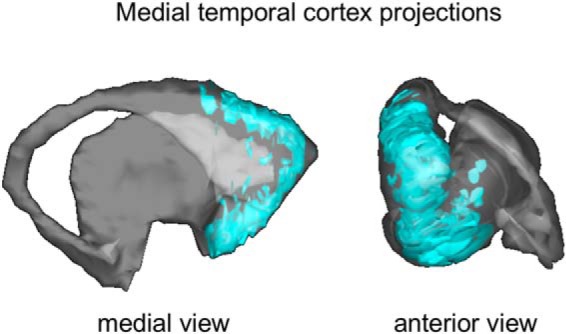
MTC projections to the striatum. 3D model of aggregate striatal projections (cyan) from anterograde injections in the anterior and posterior MTC (EC, areas 36, TH, TL/TF, and TF). Note the split in projections to the dorsal and ventral striatum and their convergence in the rostral pole of the caudate.

Area TG projects primarily medially to the VS. Specifically, terminals were located mostly along the medial wall of the caudate and in the medial and central VSd, with some scattered patches in the lateral VSd (Fig. 3*Aii*). Caudally, they extended ventrally into the VSv (Fig. 3*Aiii*). In contrast, terminals from area TEr were located more laterally in the VS, specifically in the central and lateral VSd and VSv (Fig. 3*Bii*, *Biii*). No dense patches were observed in the medial VSd nor the medial VSv. Thus, along the medial VS, there was little overlap between these projections and those from TG. Projections from both the anterior (Fig. 3*C*, *D*) and posterior (Fig. 3*E–G*) MTC terminated primarily in the VSv. Several cases also showed inputs to the VSd with generally more projections to the lateral half (Fig. 3*D*, *E*, *G*). There were no rostro-caudal topographical differences to their terminal fields, despite the rostro-caudal variation in the MTC injection sites. However, importantly, all MTC cases also showed projections to the dorsal edge of the caudate, forming a dorsal “cap” of terminals (Fig. 3*C–G*). These split MTC terminal fields were located along the dorsal and ventral edges of the striatum and converged at the rostral pole of the caudate (Fig. 3*Ci–Gi* and Fig. 4).

#### Retrograde tracer injections

**Figure 5.**
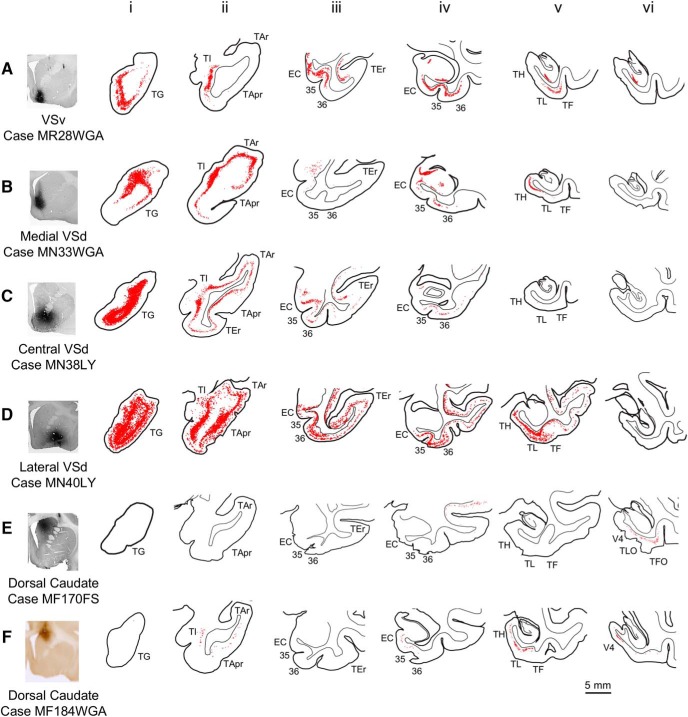
Temporal inputs to the striatum: retrograde labeling. Coronal sections of TP+ and MTC from four tracer injections in the VS (***A–D***) and two injections in the dorsal caudate (***E***, ***F***). All parts of the VS receive strong projections from the TP and MTC. Rostral inferior temporal cortex (area TEr) sends projections primarily to the VSv and central and lateral VSd.

Retrograde tracer injections in the VSv and the medial, central, and lateral VSd confirmed and extended the input patterns shown by the anterogradely labeled TP+ and MTC projections. Consistent with the TG terminals seen in all sectors of the VS, all the retrograde tracer injections labeled cells in TG (Fig. 5*Ai–Di*). The distribution of labeled cells extended caudally into TI (Fig. 5*Aii–Dii*). For all VSd injections, labeled cells were also seen in rostral auditory areas (TAr, TApr; Fig. 5*Aii–Dii*). Consistent with the primarily lateral TEr terminals, injections in the VSv and central and lateral VSd showed inputs from TEr (Fig. 5*Aiii*, *Ciii*, *Diii*), but not for the medial VSd (Fig. 5*Biii*). All VS injections labeled cells in the MTC, with the densest label seen for the VSv (Fig. 5*Aiii–Av*) and lateral VSd (Fig. 5*Diii–Dv*), as indicated by the anterograde injections. Finally, two retrograde tracer injections placed in the dorsal caudate confirmed the dorsal cap of MTC projections. Both injections showed labeled cells in the posterior MTC (TH, TL, TLO, TFO) and V4 (Fig. 5*Evi*, *Fv*, *Fvi*). Interestingly, one injection also labeled cells more anteriorly in the anterior MTC (EC, area 35) and TP+ (TI, TApr; Fig. 5*Fii*, *Fiv*).

### Convergence of frontal and amygdala projections with temporal polar and medial temporal projections in the ventral striatum

#### Anterograde tracer injections

**Figure 6.**
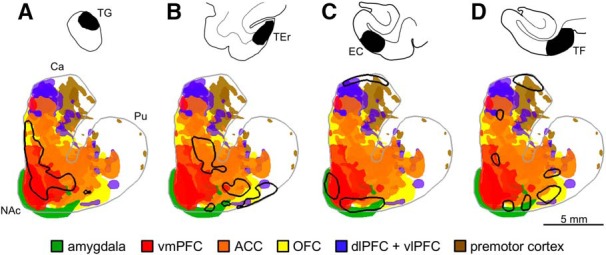
Overlap of temporal, frontal, and amygdala projections in the striatum. Outlines (black) of dense projections from TP+ and MTC are overlaid on schematic composites of dense projections (filled) from various frontal regions. Temporal injection sites shown above. Ca, caudate; Pu, putamen; NAc, nucleus accumbens.

Details of frontal and amygdala projection patterns have been previously reported (Künzle, 1978; Russchen et al. 1985; Selemon and Goldman-Rakic, 1985; Haber et al. 1995, 2006; Ferry et al. 2000; Fudge et al. 2002; Calzavara et al. 2007). Briefly, these prior findings showed that PFC and amygdala projections form a general ventromedial-to-dorsolateral gradient of inputs (Fig. 6). The basal and central nuclei of the amygdala target the VS (Russchen et al. 1985; Fudge et al. 2002). Projections from the vmPFC (areas 14, 25) also terminate in the VS and along the medial wall of the caudate. OFC (areas 11, 13) projections overlap with vmPFC terminals and extend further dorsolaterally into the VSd and dorsal caudate. ACC (area 24) inputs also span the VSv, VSd, and dorsal caudate, forming a more lateral territory that overlaps with the OFC territory. dlPFC (areas 9, 46, 9/46, 8) and vlPFC (areas 47, 44/45) inputs terminate further dorsolaterally in the dorsal caudate with smaller patches in the VSd. Premotor projections are located adjacent to these dlPFC inputs, primarily within the dorsolateral caudate and putamen.

Comparison of these frontal and amygdala projections with the present temporal projections suggested two broad convergence patterns in the VS. The first pattern spanned the ventromedial VS, comprised of the medial VSd and the VSv. Here, TG projections converged specifically with the inputs from the vmPFC, including along the medial wall of the caudate (Fig. 6*A*). These projections also overlapped with ACC, OFC, and amygdala inputs in the medial VSd and VSv (Fig. 6*A*). The second convergence pattern involved the dorsolateral VS composed of the central and lateral VSd. Here, in contrast to TG and vmPFC projections, TEr projections overlapped with dlPFC and vlPFC inputs, as well as with those from the ACC, OFC, and amygdala (Fig. 6*B*). Like amygdala projections, EC and TF terminals from the MTC were found in both the VSv and VSd and overlapped with inputs of both convergence patterns (Fig. 6*C*, *D*). In addition, these MTC projections also converged with dlPFC, vlPFC, and premotor inputs in the dorsal cap of the caudate (Fig. 6*C*, *D*).

#### Retrograde tracer injections

**Figure 7.**
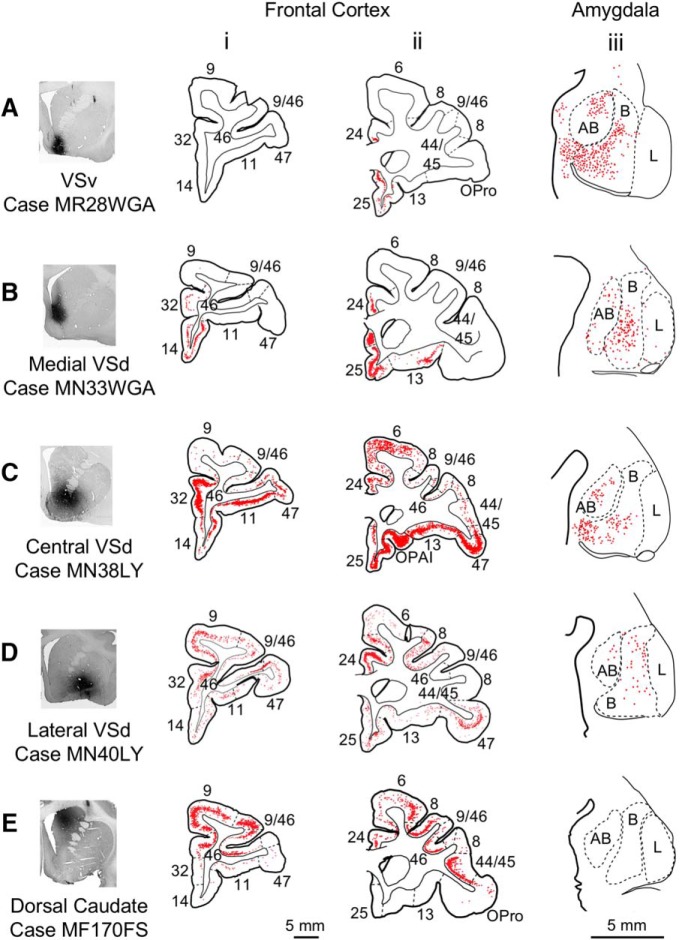
Frontal and amygdala inputs to the striatum: retrograde labeling. ***A–D***, Four retrograde tracer injections in VS. ***E***, One injection in the rostral dorsal caudate. Coronal sections of the frontal cortex (***i***, ***ii***) and amygdala (***iii***). AB, accessory basal nucleus; B, basal nucleus; L, lateral nucleus. ***Aiii***, ***Ciii***, and ***Diii*** are reprinted with minor stylistic edits from Fudge et al. (2002) with permission from Elsevier, Inc.

The retrograde VS injections confirmed the two broad convergence patterns in the ventromedial and dorsolateral VS and identified the comprehensive set of TP+, MTC, frontal, and amygdala inputs to each VS region. Consistent with the ventromedial VS pattern, in addition to inputs from TG, TI, and MTC, the VSv (Fig. 7*Aii*) and medial VSd (Fig. 7*Bi*, *Bii*) received afferents from ventral and medial PFC regions. Collectively, these were located in the vmPFC (areas 14, 25), ACC (areas 24, 32), and caudal OFC (area 13). Labeled cells were also seen in the basal and accessory basal nuclei of the amygdala (Fig. 7*Aiii*, *Biii*). In contrast, retrograde tracer injections in the central (Fig. 7*Ci*, *Cii*) and lateral (Fig. 7*Di*, *Dii*) VSd confirmed the different combination of inputs forming the dorsolateral VS pattern. In addition to temporal inputs from across TP+, including TEr, and MTC, the central and lateral VSd received projections from both ventral and medial PFC that contribute to the ventromedial VS, as well as frontal polar (area 10) and dorsal and lateral PFC (9, 46, 9/46, 8, 44/45, and 47) regions. Inputs from the basal amygdala also targeted the central and lateral VSd (Fig. 7*Ciii*, *Diii*). Finally, in contrast to the VS, retrograde tracer injections involving the dorsal caudate cap targeted by MTC projections showed labeled cells primarily in the frontal pole (area 10), dorsal and lateral PFC (areas 9, 46, 9/46, 8, 44/45), and premotor cortex (area 6; Fig. 7*Ei*, *Eii*), as expected from frontal anterograde projection patterns. No labeled cells in the amygdala were observed (Fig. 7*Eiii*), consistent with known amygdala projections to the ventral, but not dorsal, striatum.

**Figure 8.**
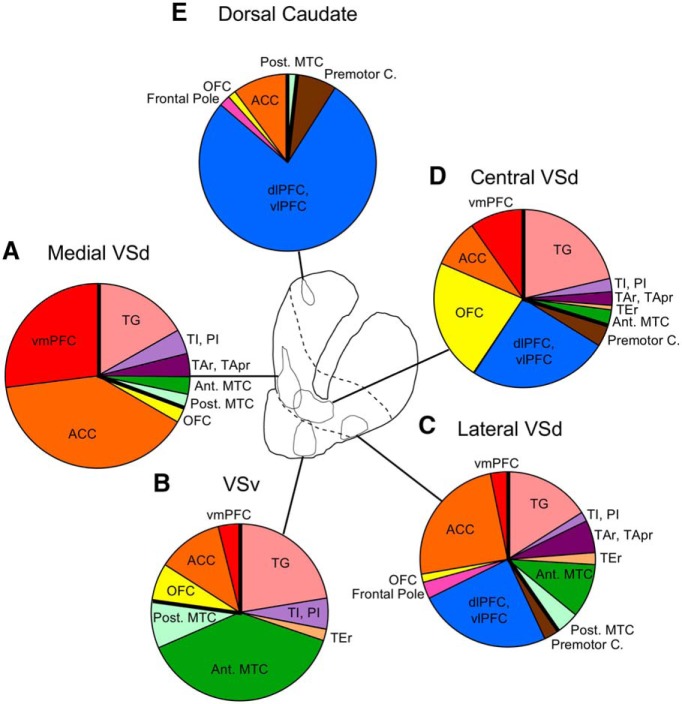
Relative contributions (percentage) of TP+, MTC, and frontal cortical inputs to the striatum. Thick black lines separate temporal and frontal cortical areas. See Table 2 for percentage values. Same cases as in Fig. 7.

Quantification of the labeled cells within TP+, MTC, and frontal cortical areas from each VS injection and a dorsal caudate injection (Case MF170FS) specified the relative strengths of their inputs to the different VS sectors (Fig. 8; Table 2). As described above, the medial VSd (Fig. 8*A*) and VSv (Fig. 8*B*) both received inputs from TP+, MTC, and ventral and medial PFC regions, including the vmPFC, ACC, and OFC. In the lateral (Fig. 8*C*) and central VSd (Fig. 8*D*), the proportion of these inputs was altogether lower and additional inputs were present from the dlPFC, vlPFC, premotor cortex, and sensory areas of TP+. In contrast, the dorsal caudate (Fig. 8*E*) predominantly received inputs from the PFC and relatively few inputs from the posterior MTC.

**Table 2.** Relative contributions (percentage) of TP+, MTC, and frontal cortical inputs to the striatum

Region and area	VSv	Medial VSd	Central VSd	Lateral VSd	D Caud
vmPFC					
14	0	10.66	4.55	1.26	0.20
25	3.91	16.21	5.19	1.92	0.04
ACC					
24	12.05	23.12	8.17	19.00	8.82
32	0	16.31	0.60	5.50	0.65
OFC					
11, 13	0	2.48	16.41	1.53	1.41
OPAI	4.73	0	5.66	0	0.07
OPro	2.04	0	0	0	0
Frontal pole					
10	0	0.12	0.12	3.06	1.92
dlPFC					
9	0	0.20	1.91	4.48	15.77
46	0	0	1.00	1.29	14.55
9/46	0	0	0.48	2.22	11.62
8	0	0	1.14	5.70	20.53
vlPFC					
47	0	0.03	17.27	7.44	1.90
44, 45	0	0	1.94	1.42	9.45
ProM	0	0	1.68	2.10	0.37
Premotor					
6	0	0	4.01	2.86	6.91
Temporal pole					
TG	22.41	16.68	21.34	15.99	0
TI	5.65	3.92	2.08	1.68	0
PI	0	0.38	0.33	0.08	0
TAr	0	3.79	0.58	1.28	0
TApr	0.02	0.24	1.87	4.70	0
TEr	2.11	0.01	0.83	2.21	0
Anterior PHC					
EC	23.10	1.06	1.04	1.93	0
35	6.27	1.12	0.72	3.97	0
36	8.89	0.87	1.00	4.02	0
Posterior PHC					
TH	0	1.84	0	1.02	0
TL	5.04	0.41	0	2.61	0.06
TF	2.22	0	0	0.62	0.35
THO	0	0	0	0	0.19
TLO	0	0	0	0	0.85
TFO	0	0	0	0	0.24

The relative contribution of inputs from each area was calculated as the percentage of counted cells within that area versus the total number of counted cells from all frontal and temporal cortical areas examined within the same case. Same cases as in Fig. 7.

Furthermore, within these broad convergence patterns, each VS site had a unique combination of inputs (Table 2). Although both the medial VSd and VSv received inputs from the same areas, the relative proportion of inputs was different. The medial VSd (Fig. 8*A*) received inputs primarily from TG (16.7%), ACC (39.4%), and vmPFC (26.9%), whereas the VSv (Fig. 8*B*) received a lower proportion overall of PFC inputs and a higher proportion of temporal, specifically MTC (45.5%), inputs. There were also some TEr inputs (2.1%) to the VSv, consistent with the anterograde TEr projections to the lateral VSv. Within the dorsolateral VS, both the lateral and central VSd received similar proportions of TG, dlPFC, vlPFC, and premotor inputs. However, the lateral VSd received a higher proportion of ACC (24.5%), MTC (21.1%), and sensory temporal (TAr, TApr, TEr; 8.2%) inputs, whereas the central VSd received more OFC (23%) and vmPFC (10.2%) inputs.

As prior studies have noted for the ACC, OFC, and amygdala (Fudge et al. 2002; Haber et al. 2006), certain areas projected broadly to all of the examined VS sites. Of these areas, TG sent inputs roughly at the same proportion (16%–22.4%) to all sites, although from different parts of this area. Additional areas in the MTC, vmPFC, ACC, and OFC also projected to all VS sites, but at varying proportions of the overall inputs.

These differences in the distribution of inputs were reflected in the pairwise linear correlations of the areal input percentages for pairs of injection cases (Table 3). The strongest correlations were between the lateral VSd and the other VS sites (*r* = 0.53–0.69, *p* = 2.0 × 10^−5^ to 2.1 × 10^−3^, paired *t* test), consistent with the high degree of functional diversity in the inputs to the lateral VSd. The distributions of inputs for the medial and central VSd and VSv sites were also correlated with one another, but to a lesser degree (*r* = 0.36–0.41, *p* = 0.02–0.05, paired *t* test), reflecting the more site-specific inputs to these regions. None of the VS sites had distributions that were significantly similar to those of the dorsal caudate injection site.

**Table 3.** Pairwise linear correlations and *p*-values of the distributions of frontal and temporal inputs for pairs of striatal regions

Injection site 1	Injection site 2	*r*	*p* (two-tailed)
Vsv	Medial VSd	0.41	0.02
Vsv	Central VSd	0.36	0.05
Vsv	Lateral VSd	0.53	2.1 × 10^−3^
Vsv	Dorsal caudate	–0.19	0.30
Medial VSd	Central VSd	0.41	0.02
Medial VSd	Lateral VSd	0.69	2.0 × 10^−5^
Medial VSd	Dorsal caudate	–0.08	0.67
Central VSd	Lateral VSd	0.58	5.7 × 10^−4^
Central VSd	Dorsal caudate	–0.06	0.73
Lateral VSd	Dorsal caudate	0.21	0.26

Pairwise linear correlations were calculated between the distributions of input percentages from 31 frontal and temporal areas examined (see Table 2) for pairs of striatal regions. Two-tailed *p*-values were obtained with Fisher’s *r*-to-*t* transformation and Student’s *t*-distribution. Degrees of freedom = 29. Same cases as in Fig. 7.

## Discussion

### Heterogeneous map of the ventral striatum

This study examined the patterns of convergent inputs to the striatum from the temporal cortex, specifically TP+ and MTC, in relation to the well-characterized functional topography formed by frontal cortical and amygdala projections. The results demonstrate that the VS contains heterogeneous subregions that become apparent when considering both the identity and strengths of inputs, including those from regions beyond the frontal cortex. Using anterograde tracers, we found that certain temporal areas had novel projection patterns compared with frontal projections: TEr projects to the central and lateral VSd and VSv. In contrast, TG had projections located primarily along the medial wall of the caudate and extending into the central VSd and VSv, which overlapped remarkably specifically with vmPFC inputs. Using retrograde tracers, we comprehensively identified the set of inputs to specific striatal regions. With these expanded criteria, we parcellated the VS into a ventromedial sector receiving motivation and emotion-related information from areas including TG, vmPFC, ACC, and the amygdala; and a dorsolateral sector that receives this information coupled to cognitive and sensorimotor information from dlPFC, vlPFC, premotor cortex, TAr, and TEr. Thus, in general, the ventromedial VS receives the most limited inputs from regions that process information related to reward, emotion, and motivation. In contrast, the dorsolateral VS receives inputs from multiple functional areas that cut across “limbic,” cognitive, and sensorimotor domains.

Furthermore, each of these sectors could be parcellated into smaller regions that reflect differences in the proportions of their inputs. Thus, although the medial VSd and VSv both received inputs from largely the same frontal and temporal areas, they differ in the strength of those inputs: the medial VSd received a majority of inputs from the vmPFC and ACC, whereas the VSv received greater MTC inputs. The output of these two regions may therefore be related, but complementary to one another. These results underscore the importance of considering not only the source of afferents, but also their strengths when assessing the nature of the integration occurring in a region. Here, considering input strength identified additional functional heterogeneity than considering input area alone.

These results are consistent with human striatal parcellations using neuroimaging methods, which identify both an overall ventromedial-to-dorsolateral gradient (Draganski et al. 2008; Barnes et al. 2010; Bohanna et al. 2011; Tziortzi et al. 2014) and smaller subdivisions within (Cohen et al. 2008; Choi et al. 2012; Pauli et al. 2016). For example, fine-grained striatal maps based on fMRI task activity, fcMRI, or T1-weighted voxel-based morphometry show a ventromedial-to-dorsolateral gradient from limbic, to association, to motor association territory (Cohen et al. 2008; Choi et al. 2012; Pauli et al. 2016). They also show finer subdivisions that overall distinguish the ventral VS from the dorsal VS based on associations with the OFC or the dorsal medial and lateral PFC regions, respectively. The present anatomic results provide structural confirmation of these neuroimaging-based parcellations and further precisely identify the heterogeneity of inputs to distinct subregions of the VS. This anatomic map provides a guide for understanding striatal activity following task-based functional neuroimaging, as well as neurophysiological recordings.

### Potential connectional hubs in the central and lateral VSd

We previously reported the presence of connectional hubs in the striatum that receive a high degree of diverse functional inputs (Averbeck et al. 2014; Choi et al. 2017). With the limited number of anterograde injections in functionally diverse temporal areas, the present study was not suited for analyzing for hubs. However, the retrograde tracer injections in the VS indicated that the central and lateral VSd receive a high degree diverse inputs. These inputs were from motivation and emotion-related areas (TG, TI, vmPFC, OFC, ACC, amygdala). They also included inputs from cognitive (MTC, dlPFC, vlPFC) and sensory association (TAr, TApr, TEr) regions, as well as limited inputs from premotor cortex (area 6). Prior studies have reported additional projections to the central and lateral VSd from auditory association cortex in the superior temporal cortex (Yeterian and Pandya, 1998) and posterior parietal projections carrying visuospatial attentional information (Cavada and Goldman-Rakic, 1991; Yeterian and Pandya, 1993). Similarly, Luppino and colleagues reported that a distributed set of cortical regions belonging to a “lateral hand-grasping network,” including areas in the dlPFC (area 46v), vlPFC (area 12), premotor cortex (area 6V), rostral IPL (area PFG), and rostral intraparietal sulcus, sends convergent projections to the lateral VSd (Gerbella et al. 2016). This functional diversity of inputs to the central and, especially, lateral VSd indicate that they may contain critical hubs of integration. Together, the dorsolateral VS is in a position to link stimuli with cognitive states and help trigger motor programs.

### Medial temporal cortex projections to both the ventral and dorsal striatum

In a rare split of projections, all of our anterograde tracer injections placed in the entorhinal, perirhinal, and parahippocampal cortices of the MTC showed terminals in both the VS and the DS. Within the VS, these projections terminated broadly, with the strongest inputs to the VSv, and overlapped with all frontal, TP+, and amygdala afferents. In the DS, these MTC inputs formed a cap at the dorsal edge of the caudate and overlapped with dlPFC, vlPFC, and premotor projections. Interestingly, these dorsally targeting MTC inputs also overlapped with a connectional hub in the rostral dorsal caudate (Choi et al. 2017). This hub receives convergent inputs from cognitive control, reward-related, and attentional regions in the dlPFC, vlPFC, OFC, ACC, inferior parietal lobe, and area TPO of the superior temporal cortex (Choi et al. 2017). The present results show that this hub also receives inputs from the MTC, indicating the addition of memory-related information. The split in these MTC projections to the VS and DS may arise either from adjacent neurons within the MTC that separately target the VS or the DS or from single neurons that target both the VS and DS through axon collaterals. This dual striatal targeting suggests the interesting possibility that the same memory-related neural code is provided simultaneously to the VS and the DS to coordinate functionally distinct, but related, processing in both regions. Such coordinated processing may underlie the binding of multiple facets of a complex behavioral program.

### Implications for psychiatric diseases

As a central structure for reinforcement learning and habit formation, the VS has been repeatedly observed to have abnormal activity in psychiatric disorders (Robinson and Berridge, 2000; Pizzagalli, 2014; Heinz et al. 2016; Heller, 2016). By distinguishing functionally distinct subregions of the VS, this parcellation provides a guide for interpreting clinical neuroimaging results that may differentiate between healthy activations and abnormal psychiatric subtypes based on differential VS activations. The VS, often together with the adjacent internal capsule, has become one of the major lesion and investigational deep brain stimulation (DBS) targets for refractory cases of obsessive-compulsive disorder, depression, and addiction (Greenberg et al. 2010; Müller et al. 2013; Cleary et al. 2015). DBS, in particular, which has become established as a highly effective, reversible treatment for movement disorders such as Parkinson’s disease, is being investigated to treat an increasing number of psychiatric conditions. However, unlike its efficacy in movement disorders, DBS of the VS has seen partial response in psychiatric cohorts (Morishita et al. 2014; Cleary et al. 2015). Part of this may be related to the selection of the target site. The present map indicates that the VS is a complex area divisible into quite different partitions based on varying overlaps of projections from diverse cortical and subcortical regions. Targeting a given VS site stimulates projections not simply from a single or a few cortical regions, such as the OFC or ACC, but rather the selective striatal interactions of multiple cortical and subcortical regions. This anatomic VS parcellation provides a map for optimally targeting precise sites and inputs pertinent to specific disease conditions.

## Conclusions

This study builds on the well-characterized functional map of the striatum based on frontal cortical and amygdala projections. We present an updated, heterogeneous map of the VS by considering both the identity and strengths of the inputs received, including those from regions beyond the frontal cortex, specifically TP+ and MTC in the temporal cortex. The VS contains a ventromedial sector receiving motivation and emotion-related information from areas including TG, vmPFC, ACC, and the amygdala; and a dorsolateral sector that receives this information coupled to cognitive and sensory information from dlPFC, vlPFC, premotor cortex, TAr, and TEr. Each sector further contains distinct smaller regions that receive different proportional strengths of inputs, suggesting that each region of the striatum has a unique combination and integration of inputs. These results indicate that the striatum contains a complex, selective combination of inputs, providing the anatomic substrates for forming myriad functional associations that add to network interactions in the cortex. This heterogeneous parcellation of the VS provides a map of this combinatorial integration. This map can be used in guiding and interpreting healthy and abnormal striatal activity in normal subjects and those with mental health disorders and for targeting specific functional interactions in different disease conditions.

## Synthesis

Reviewing Editor: Menno Witter, Norwegian University of Science and Technology Decisions are customarily a result of the Reviewing Editor and the peer reviewers coming together and discussing their recommendations until a consensus is reached. When revisions are invited, a fact-based synthesis statement explaining their decision and outlining what is needed to prepare a revision will be listed below.

This is a transferred paper from J. Neuroscience witha clear and positive recommendation. The author shave adequately responded to the sugegstions and remarks brought forward by the two reviewers. The paper in its present form is acceptable when the authors make three additional minor changes.

In line 138 please add field following light- and darkfield microscopy.

In that same section the authors refer to 1 in 8 and 1 in 24 sections. Although most anatomists might know what this means, the general reader might misunderstand, so please clarify.

Again in the same section, the authors write that stereoinvestigator was used to identify labeled cells in areas of the brain. I do not think that is correct. The authors identify areas and labelled neurons, stereoinvestigator allows to obtain an unbiased quantitavie estimate of the number of cells/volume or surface. Please rewrite.

## Author Response

We, the authors, thank the editor and reviewers for their rapid and helpful feedback on our manuscript. We have addressed each point, below.

Synthesis of Reviews:

Computational Neuroscience Model Code Accessibility Comments for Author (Required):

N/A

Significance Statement Comments for Author (Required):

N/A

Comments on the Visual Abstract for Author (Required):

N/A

Synthesis Statement for Author (Required):

This is a transferred paper from J. Neuroscience with a clear and positive recommendation. The authors have adequately responded to the suggestions and remarks brought forward by the two reviewers. The paper in its present form is acceptable when the authors make three additional minor changes.

In line 138 please add field following light- and darkfield microscopy.

We have made this change.

In that same section the authors refer to 1 in 8 and 1 in 24 sections. Although most anatomists might know what this means, the general reader might misunderstand, so please clarify.

We have made this clarification in this paragraph and the previous paragraph.

Again in the same section, the authors write that stereoinvestigator was used to identify labeled cells in areas of the brain. I do not think that is correct. The authors identify areas and labelled neurons, stereoinvestigator allows to obtain an unbiased quantitavie estimate of the number of cells/volume or surface. Please rewrite.

We have corrected and rewritten this part.
